# Measuring Bee Effects on Seed Traits of Hybrid Sunflower

**DOI:** 10.3390/plants12142662

**Published:** 2023-07-16

**Authors:** Gary J. Brewer, Kentaro Miwa, Kathryn Hanford

**Affiliations:** 1 Department of Entomology, University of Nebraska-Lincoln, Lincoln, NE 68588, USA; 2 Department of Statistics, University of Nebraska-Lincoln, Lincoln, NE 68588, USA; kathy.hanford@unl.edu

**Keywords:** pollination ecology, exclusion cages, yield, seed set, integrated pest management

## Abstract

In hybrid sunflower, bee pollination can improve productivity, but the contribution of bees to productivity may be over or underestimated. To estimate bee effects (seed trait gains from exposure to bees during anthesis), single capitula are commonly covered with a porous material to exclude bees. However, depending on the exclosure porosity, estimates of the magnitude of bee effects will vary. In two studies, porosity size and bee effect gains in two sunflower types were tested. In the exclosure study, Delnet exclosures severely reduced seed set and exclosures with larger porosities and had smaller and similar effects. However, since a few small bees penetrated the largest porosity size tested, exclosures with porosity sizes < 7 mm are recommended. With an exclosure porosity of 5 X 5 mm, the estimated bee effect contribution to the yield was 323 kg per hectare. Effects of exclosures on seed traits were similar in the oilseed and confectionary hybrids tested. Insecticide use did not affect seed traits but did lower insect damage to seeds. Bees from three families, mostly Apidae, were collected while foraging on sunflower. In summary, we recommend the use of exclosures with porosities of about 3 to 5 mm to avoid over or underestimating bee effects. And we recommend holistic insect management for sunflower cropping systems that balances the benefits of bee effects on seed traits with management of pest insects.

## 1. Introduction

The sunflower, *Helianthus annuus* L., (Asterales: Asteraceae) is an important annual crop in the United States and globally [1]. Sunflowers are attractive to various insects, including pest and beneficial species. Numerous bees forage sunflowers during anthesis [2] and are important in sunflower pollination. Although wild sunflower is self-incompatible [3,4], sunflower hybrids have been bred to be self-compatible [5] and can set seed without insect cross-pollination [6,7].

To better understand the pollination ecology of hybrid sunflower, a variety of approaches, mainly bee exclusion methods, have been used to investigate bee effects, the result of bee cross-pollination on seed variables. Moreti et al. [8] used multi-plant cages and single capitula (sunflower head) bag exclosure techniques to control bee access to flowering sunflower and found that seed number and weight were increased by bee access. Vishwakarma and Ghaatak [9] found that multi-plant cage studies with honey bees (*Apis mellifera* L., Hymenoptera: Apidae) produced the highest yield compared to other pollination systems. Most studies performed using exclusion techniques found that seed productivity was increased when honey bees or wild bees had access to sunflower heads [8,10,11,12,13,14]. In a landscape study, Bartual et al. [15] found that sunflower seed set was increased when surrounding areas had an abundance of honey bee hives and early flowering crops. Seed oil concentration, a yield component important to growers of oilseed sunflower, has also been reported to increase when sunflowers were pollinated by bees [15,16,17,18,19].

However, other studies have reported none or variable bee effects with sunflower hybrids open to bees. Astiz et al. [20] tested two oilseed sunflower hybrids using a bag technique and did not observe yield improvement associated with pollinators, and inferred that the test hybrids had high self-compatibility. In another bag exclusion study, Mallinger and Prasifka [21] investigated bee effects on the yield of confection sunflower hybrids and found that only five of the fifteen hybrids tested had yield gains when open to bees. The variation in bee visitation rates and degree of self-fertility they observed may partly explain why only some hybrids expressed a yield gain with bee exposure. Similarly, a 50-hybrid study found that most but not all hybrids tested had increases in seed set, seed weight, and seed oil percentage when open to bee pollination [22].

Besides the value bees bring to sunflower production, bees also benefit from the interaction, as sunflower can be a valuable forage crop. Honey bees are the only managed bees used in sunflower [23] and can be the main pollinator found in commercial sunflower fields [24,25]. However, in other studies, wild bees were more abundant than honey bees on sunflower [21].

A diversity of wild bee species forage sunflower to collect pollen and nectar [2]. Bombini and Anthophorini bees (Family Apidae) are commonly found in commercial sunflower in North Dakota [25]. Insects other than bees also pollinate sunflowers [26,27,28,29]. Although wind is not considered a major factor in sunflower pollination [26,30], in some studies wind pollination has been estimated to contribute from 20 to 27% of seed set [31,32].

Insect seed predator guilds also directly affect sunflower seed productivity by eating or killing seeds [33,34]. In North Dakota and Nebraska USA, where our studies were conducted, the major seed predators that co-occur with bees on flowering sunflower are the red sunflower seed weevil (*Smicronyx fulvus* LeConte, Coleoptera: Curculionidae), the sunflower moth (*Homoeosoma electellum* (Hulst), Lepidoptera: Pyralidae), and the banded sunflower moth (*Cochylis hospes* Walsingham (Lepidoptera: Cochylidae) [24].

Cultural and biological controls can be used to manage insect seed predators in sunflower, but insecticides are used almost exclusively and are effective when used appropriately [24,35,36]. To manage seed insects, insecticides are applied during anthesis [36]. A potential outcome of insecticide use can be a decline in bee population levels and the reduction or loss of the benefits of bee cross-pollination, i.e., bee effects [37,38].

Because honey bees and wild bees co-occur on sunflower during anthesis and because previous work has documented that bee populations can increase commercial sunflower yield, accurate assessment of bee effects in various locations and production systems is needed. The objective of this study was to compare exclosure material porosity to improve accuracy in estimation of bee effects. Anticipated outcomes include more accurate estimates of bee effects and the development of sunflower production guidelines that include estimation of bee effect gains and promoting protection and conservation of bee populations.

## 2. Results

### 2.1. Insects

Honey bees and endemic populations of wild bees and seed feeding predators were present in both studies. Banded sunflower moth and red sunflower seed weevil were the major seed feeding insects in the exclosure study, and in the hybrid comparison study, sunflower moths were abundant and small numbers of red sunflower seed weevils were present.

Bees from three families were collected foraging on capitula with most species in the family Apidae (Table 1). Representative voucher specimens of the bees collected were deposited in the University of Nebraska State Museum.

### 2.2. Bee Exclusion Study

Mean number of seeds per capitula increased as access to bees increased. Similarly, yield (grams/capitula) increased significantly with increasing bee access (Table 2). Delnet^®^ pollination bags with the smallest porosity size of the exclosures tested and the most restricted bee access resulted in the largest reductions in seed set and yield.

The bee effect gains for the Mesh-1 exclosure were increases of 232 seeds (1368 minus 1136) and 8.6 g (75.2 minus 66.6) per capitula. Using the bee effect yield gain of 8.6 g from the Mesh-1 exclosure and assuming a plant population of approximately 37,500 per hectare, the yield increase would be 323 kg per hectare. At market prices of 0.49 to 0.53 USD per kg, bee effect gains would be valued from 158 to 170 USD per hectare.

No bees were observed when accessing florets through the Delnet^®^ or Mesh-1 exclosures during visual observations. However, a few small bees were able to penetrate the Mesh-2 exclosures.

### 2.3. Hybrid Study

Because of the high sunflower moth populations in the hybrid study, plants treated with insecticide were sprayed three times a week throughout the flowering period. Insecticide use did not cause observable effects on bee visitation, as bees were present on heads the day after application. Mesh bags were largely effective in excluding bees, although, some small bees, such as *Halictus* spp., were occasionally found inside the mesh bags. Insects smaller than bees were not excluded by the mesh bags.

Exclosure use reduced seed set and yield for both hybrids. However, insecticide use was less consistent and improved seed set only for the oilseed hybrid. Insecticide use did reduce insect injury to a small extent for both hybrids. Yield per capitula was highest when exclosures were not used and was unaffected by insecticide use. However, yield adjusted to a per area basis (cm^2^) was increased by insecticide use (Table 3) suggesting that yield per capitula area may be a more sensitive measure than yield per capitula.

Seed oil percentage, an important quality trait for oilseed sunflower was highest when bees had access to the sunflower florets, i.e., exclosures were not used. Despite multiple insecticide applications, insecticide use did not result in large decreases in insect damage to seeds, and exclosures increased insect injury to seed (Table 3).

Interaction effects were not significant, except for seed damage to the oilseed hybrid. However, for both hybrids, the interaction of no exclosure use (i.e., bees had access to florets), and insecticide applied numerically, increased seed set and reduced seed damage (Table 3). For hybrid Seeds 2000 Blazer, the oilseed hybrid, seed oil percentages were highest in the two exposure groups without exclosures (bee access allowed). For the other seed traits, notable interaction effects were not observed.

## 3. Discussion

### 3.1. Bee Exclusion Study

Wild sunflowers are dependent on insect pollination to set seed [3,4] but hybrid sunflower, as discussed earlier in this paper, has been bred to be self-compatible [6,7]. However, despite hybrid’s self-compatibility, there is evidence that sunflower yield and other traits can be improved by the presence of bees [8,10,11,12,13], even though some reports show little or no benefit [20,21]. To understand how bees increase sunflower production, yield and other traits, potential bee effect gains need to be accurately determined.

Bee exclusion experiments are a type of cage study, and they risk biasing the results with confounding factors [39,40]. Luck et al. [41] urged that data from cage trials should be used with caution as they may not represent true experimental results [42]. Typically, bee effect studies use materials with sufficient porosity to allow transpiration and excess moisture to escape and that is small enough to prevent bees from accessing the sunflower florets. However, the porosity of the materials used is often not reported. This study reports on cage effects found in single capitula, bee exclusion studies in sunflower.

Seed set in hybrid sunflower results from a combination of inherent self-pollination, cross-pollination by bees and other insects, and by some wind pollination. Bee effect studies aim to remove the contribution of bee pollination and measure all other pollination sources. Trait differences (seed set, yield, etc.) between open and bee excluded capitula are the contribution bees make to the seed traits, i.e., bee effects.

Attempts to measure bee effects with exclosures with very small opening (porosity) sizes will exclude almost all potential insect cross-pollination and prevent wind pollination, thus, overestimating bee effects. However, if the exclosure material’s porosity is too large, pollination by other insects and by wind will appropriately be included but some bee pollination may also occur, thus bee effects could be underestimated. Ideally the exclosure material will prevent bee pollination, and allow pollination from smaller insects and wind pollination to occur.

To measure bee effects while allowing pollination by other sources, we recommend using single capitula mesh exclosures with openings of about 3 × 3 to 5 mm × 5 mm in size. Exclosures in that size range were observed to prevent bee access to the sunflower florets and had sufficient porosity to allow small insects to pass through and to allow wind pollination to occur.

### 3.2. Hybrid Study

The hybrid study used exclusion cages of moderate porosity (3 mm × 3 mm) to determine bee effects in an oilseed and a confectionary sunflower hybrid. Our results showed seed trait gains from cross-pollination and, similar to other studies, highlighted differences in how individual hybrids respond to the presence of bees during the anthesis period. We also note that sunflower responses to the concurrent presence of bee and seed predator guilds, with respect to sunflower seed traits, are not a simple gain or loss response but are, in fact, a complex interaction that needs further study.

Insecticide application did not provide the reduction in insect damaged seed, as was anticipated. The poor insecticide efficacy may have occurred because insecticides were applied to individual plants, not the whole plot. Thus, the general population of moths would largely escape treatment and be available to oviposit on the treated plants, and the resulting larvae would then increase the levels of insect injured seeds. Interestingly, exclosures increased insect injury to seed, perhaps by inhibiting natural enemy effectiveness.

Although sunflower hybrids have been bred to be self-compatible, bee effects can improve yield parameters, and production systems should be designed to promote bee activity in sunflower fields. Wild bees are efficient sunflower pollinators [43,44,45] but their populations are in decline [46,47] and may often be too low to ensure vigorous cross-pollination. Managed honey bees can be used to supplement pollination by wild bees, but honey bee pollination does not fully compensate for loss of wild bee diversity and abundance [48]. However, studies have shown that interactions between wild bees and honey bees increase overall pollination services in sunflower [43,49] and suggest a potential managed-wild bee pollination strategy for sunflower. Comprehensive pollination strategies being developed or promoted for soybean [50], oilseed rape [51], and field hedgerows [52] can serve as models for similar sunflower crop management strategies.

Our findings include a recommendation of using single plant exclosures with porosities of about 3 to 5 mm in diameter to best measure bee effects. We also emphasize the need for sunflower production systems that conserve and protect bee populations. Sunflower cropping systems that include robust bee conservation and protection practices will benefit from bee effects with increased sunflower seed productivity, and beekeepers will benefit by access to abundant foraging to increase honey production.

During the flowering period, sunflower insect management should integrate bee conservation and protection plans to enhance bee effect productivity gains that are balanced with efforts to reduce losses from insect seed predators. Additionally, sunflower integrated pest management programs need to evolve to incorporate bee effect gain values into dynamic economic thresholds. It is also clear that bee, seed predator, and sunflower interactions affecting productivity are complex and need continued investigation.

## 4. Materials and Methods

### 4.1. Foraging Bee Collections

Bees were collected by hand at 0900, 1200 and 1500 h 3 times per week during the flowering period to obtain a representative survey of bees foraging on sunflower. Specimens were identified to genus and to species (where possible) using keys and descriptions from several sources [53,54,55,56,57] and were then compared with specimens in the University of Nebraska State Museum to confirm identifications.

### 4.2. Seed Handling and Trait Assessments

At plant stage R9 (physiological maturity) [58], plants of each experimental unit (capitula) were hand-harvested and dried, and then hand-threshed and cleaned to remove debris and unfilled seeds (pericarps lacking a developed embryo). The remaining filled seeds were used to measure the following seed traits (response variables) on a per capitula basis in one or both of the field studies.

- Seed Set—filled seeds (rounded from side-to-side) with an obvious developing embryo internal to the pericarp (hull).
- Yield—grams of filled seeds.
- Capitula Area—calculated as area of a circle from two radial measures at right angles across the capitula.
- Yield per cm^2^—yield per area of capitula.
- Seed Oil—seed oil percentage was determined by a nuclear magnetic resonance analyzer, according to published methods [59].
- Insect Injury—proportion of filled seeds with evidence of insect feeding were determined according to Peng and Brewer [60].
- Bee Effect Gains were the difference of the None exclosure treatment minus the values of the exclosure treatments.

### 4.3. Bee Exclusion Study

Bag exclosures, providing various levels of access by bees, other insects, and windblown pollen to sunflower capitula from very limited to full access, were tested to determine effects on seed traits. Three porosity levels of single plant exclosures were established by securing bags with increasing pore or mesh sizes over sunflower capitula. Bag types were Delnet^®^ pollination bags (DelStar Technologies Inc., Middletown, DE, USA, pore size 0.1 to 0.14 mm) and two types of plastic, mesh grocery bags with openings of about 5 mm × 5 mm and 10 mm × 7 mm. The meshes excluded most bees but allowed seed feeding insects to pass through, and increased airflow and wind pollination compared to Delnet^®^ bags. Checks were capitula without an exclosure. Exclosures were installed on plants just prior to the start of anthesis and remained on the plants until anthesis ceased, typically about seven days.

Sixty plants at early floral bud stage were selected and tagged (15 randomly assigned per treatment) on one side of a commercial oilseed sunflower field near Kindred, North Dakota, USA. However, based on previous experience, we anticipated losses of replicates from causes such as breakage, capitula injury from abiotic and biotic causes, and uneven plant growth. At harvest, replicates per treatment ranged from 7 to 11 capitula.

### 4.4. Hybrid Study

Combinations of a bee exclusion bag (mesh opening 3 mm diameter) and insecticides were used to control the presence of bees and seed predators on sunflower capitula. See Table 4 for the combination treatment details. Treatments were randomly assigned to healthy plants, i.e., plants without visible damage or atypical phenotypes. Exclosures were placed on plants according to the methods in the Exclosure Study.

Testing was conducted at the University of Nebraska, Eastern Nebraska Research and Extension Center, NE, USA. Confection hybrid Mycogen 838A (Mycogen, Indianapolis, IN, USA), and Seeds 2000 Blazer, a NuSun oilseed variety (Seeds 2000, Breckenridge, MN, USA), were planted in 12 20 m rows. The test hybrids were selected as typical confection and oil seed hybrids without any prior knowledge of their responses to bee pollination.

The four insect exposure treatments specified in Table 4 were applied to plants in four central rows of the twelve row plots for both hybrid plots. Treatments were randomly assigned to four plants in a row. Thus, 16 plants per row were selected to receive treatment. Following the anthesis period, paper bags were placed on all test capitula to limit bird feeding on seeds. Exclosures were applied as in the Exclusion Study.

Several applications of a pyrethroid (esfenvalerate, 0.0033%, Ortho^®^ Bug-B-Gon^®^ Max^®^ Garden & Landscape Insect Killer, ORTHO Group, Marysville, OH, USA) insecticide were applied throughout anthesis to remove seed feeding insects. Applications of the pyrethroid insecticides were made late in the afternoon to minimize direct application to bees. Pyrethroids are considered to have insect repellent activity, but bee populations did not seem to be diminished one day after treatment (Brewer, personal observation).

### 4.5. Statistical Analysis

SAS Version 9.4 for Windows was used for all data analyses. The GLIMMIX procedure was used for all analysis of variance (ANOVA). An alpha level of 0.05 was used for determining significance. The MEANS procedure was used to determine means and standard errors. For the exclosure insecticide factorial study, the seed traits were analyzed using a linear model with fixed exclosure, insecticide, and exclosure by insecticide effects and the random block effect. If the interaction effect was significant, then a Tukey–Kramer adjustment was used for comparison among the means. For the exclosure study, seed traits were analyzed using a linear model with a fixed exclosure effect. A Tukey–Kramer adjustment was used for comparison among the means.

## Figures and Tables

**Table 1:** Bee families and species identifications of specimens collected by hand from individual capitula in test plots.

Families	Species
Andrenidae	*Protandrena* spp.
Apidae	*Apis mellifera* L.
	*Bombus griseocollis* (DeGeer)
	*Bombus impatiens* Cresson
	*Bombus pensylvanicus* (DeGeer)
	*Diadasia enavata* (Cresson)
	*Melissodes agilis* Cresson
	*Melissodes bimaculata* (Lepeletier)
	*Melissodes coreopsis* Robertson
	*Melissodes desponsa* Smith
	*Melissodes trinodis* Robertson
	*Svastra obliqua* (Say)
	*Triepeolus* spp.
Halictidae	*Agapostemon* spp.
	*Dieunomia* spp.
	*Halictus ligatus*

**Table 2:** Effect of single plant bee exclosures with porosities providing variable bee access to florets during anthesis on mean (SE) of sunflower seed traits and bee effect gains per capitula and ANOVA statistics.

Exclosure	Porosity	Degree of			Yield	Bee Effect Gains ^3^	
Treatment	(mm)	Bee Access ^1^	N ^2^	Seed Set	(Grams)	Seed Set	Yield
Delnet^®^	0.0–0.14	Excluded	10	826 (51) c	57.1 (3.6) b	542 (70)	18.1 (5.0)
Mesh-1	5 × 5	None seen	7	1136 (61) b	66.6 (4.3) ab	232 (78)	8.6 (5.5)
Mesh-2	10 × 7	Limited	10	1090 (51) b	76.1 (3.6) a	278 (70)	−0.9 (5.0)
None	-	Unlimited	11	1368 (48) a	75.2 (3.5) a	-	-
F				19.97	6.04		
df				3.34	3.34		
*p*				<0.0001	0.0021		

Means in a column with different letters are significantly different at *p* = 0.05 alpha level, based on Tukey’s multiple comparison adjustment. ^1^ Bee access to sunflower capitula during anthesis. None seen—no bees observed accessing florets through the mesh material. Limited—a few small bees observed inside the exclosure. ^2^ N—number of harvested replicates per treatment. ^3^ Bee Effect Gains. Means of Unlimited bee access—means from exclosures (SE of the difference).

**Table 3:** Main and interaction effects of bee exclosure and insecticide treatments on LSmeans of seed traits per capitula in a 2008 Nebraska, USA study of two sunflower hybrids and ANOVA *p*-values.

Treatments Used ^1^		Seed Set ^2^	Yield (g)	Capitula (cm^2^)	Yield /(cm^2^)	Seed Oil %	Insect Injury ^3^
Ex	I						
Main Effects		**Mycogen 838A (confection hybrid)**					
No		1074 a	126 a	350	3.1 a	28.3 a	11.a
Yes		945 b	114 b	362	2.6 b	25.0 b	20.0 b
	No	975	119	357	2.7 b	28.2 b	17.1 b
	Yes	1044	121	355	2.9 a	25.2 a	13.9 a
Main Effect SEM		33.4	4.5	11.1	0.05	0.40	0.87
Interaction Effects							
No	Yes	1113	130	352	3.2	26.6	9.0
No	No	1035	123	348	3.0	30.1	13.0
Yes	Yes	976	111	358	2.7	23.8	18.9
Yes	No	914	116	365	2.5	26.3	21.2
Interaction SEM		45.9	5.9	15.1	0.07	0.46	1.17
Sources of Variation *p*-values		*p*-values					
Exclosure		0.0067	0.0251	0.4397	<0.0001	<0.0001	<0.0001
Insecticde		0.1276	0.7968	0.9076	0.0015	<0.0001	0.0078
Ex × I		0.8618	0.2542	0.7493	0.8514	0.1658	0.4608
Main Effects		**Seeds 2000 Blazer (oilseed hybrid)**					
No		2182 a	129 a	367	5.9 a	40.7 a	15.2 a
Yes		1598 b	106 b	346	4.6 b	37.6 b	28.4 b
	No	1761 b	116	361	4.8 b	38.8	28.3 b
	Yes	2019 a	120	352	5.7 a	39.5	15.3 a
Main Effect SEM		82.8	4.2	11.0	0.15	0.46	1.19
Interaction Effects							
No	Yes	2273	132	368	6.2	41.3	10.6 a
No	No	2091	127	366	5.7	40.2	19.7 b
Yes	Yes	1765	108	336	5.2	37.7	20.0 b
Yes	No	1432	105	356	4.0	37.5	36.9 c
Interaction SEM		116.8	5.7	15.5	0.21	0.52	1.66
***Sources of Variation***		*p*-values					
Exclosure		<0.0001	0.0002	0.1824	<0.0001	<0.0001	<0.0001
Insecticde		0.0341	0.4949	0.5499	0.0002	0.0573	<0.0001
Ex × I		0.5235	0.8898	0.4733	0.1105	0.2278	0.0229

^1^ Treatments Used. Ex—Exclosure, I—Insecticide. Refer to Table 4 for details. ^2^ Seed Set—seeds per capitula. ^3^ Insect Injury—percent of seed per capitula with insect feeding injury. LSMeans within a column and for exposure groups lacking a common letter differ (*p* ≤ 0.05). LSMeans without following letters are not significantly different. Numerator df = 1, error df = 32.

**Table 4:** Hybrid study insect exposure groups (treatments), protocol combinations, and study objectives in the Nebraska hybrid study.

Exposure	Treatments Used ^1^		
Group	Exclosure	Insecticide ^2^	Pollination and Other Objectives
Bees	No	Yes	Bee and wind pollination allowed.
			Reduced seed predator and other insect
			numbers.
No Bees	Yes	No	No bee pollination. Self-pollination and
			pollination by small insects and wind
			allowed. Seed predators present.
All	No	No	Normal, no exclusions. Pollination from all
			sources. Seed predators uninhibited.
None	Yes	Yes	Self and wind pollination. Reduced seed predation.

^1^ Treatments used to create Exposure Group, bee exclosure and insecticide application. ^2^ Pyrethroid (esfenvalerate).

## Data Availability

Data is available on request from corresponding author.
